# Coexistence of Severe Valvular Pulmonary Stenosis and Papillary Thyroid Carcinoma: A Rare Association or Coincidence?

**DOI:** 10.7759/cureus.92607

**Published:** 2025-09-18

**Authors:** Mohammed R Alshehri, Ahmed M Hamad, Ramez M Othman, Omar A Shahball

**Affiliations:** 1 Pulmonary Medicine, King Fahad Medical City, Riyadh, SAU; 2 Internal Medicine, Pulmonary Department, Prince Mohammed Bin Abdulaziz Hospital, Riyadh, SAU

**Keywords:** carcinoma thyroid, papillary carcinoma of thyroid, papillary thyroid carcinoma (ptc), ps:pulmonary stenosis, pulmonary valve stenosis, syndrome or coincidence

## Abstract

Papillary thyroid carcinoma (PTC) is the most common thyroid malignancy. Pulmonary valve stenosis (PVS), on the other hand, is a congenital valvular heart disease characterized by narrowing of the right ventricular outflow tract, which can lead to right-sided heart failure if severe. To date, there are no reported cases in the literature describing a co-occurrence of PTC and PVS in the same individual. Our patient was a 28-year-old male, a smoker with a five-pack-year history, who presented with a painless right neck swelling of five months duration. Physical examination revealed a firm, non-tender right cervical lymphadenopathy. Thyroid examination showed that the trachea was midline, thyroid was non-palpable, without tenderness, nodules, or bruit, and no signs of exophthalmos or proximal muscle weakness. Heart examination revealed a thrill, a normal first and second heart sounds, and a systolic crescendo-decrescendo murmur heard at the left upper sternal border. Jugular venous pressure (JVP) was not elevated. No signs of ascites, hepatomegaly, or lower limbs edema. The patient had no syndromic features. Laboratory wise was unremarkable. In regard to imaging, neck ultrasound showed multiple enlarged heterogeneous rounded structures in the right cervical region, the largest measures 2.9 x 2.5 cm. The visualized parts of the thyroid gland appeared unremarkable. Computed tomography (CT) of the brain, chest, abdomen, and pelvis was unremarkable apart from cardiomegaly in the CT chest. Further assessment by echocardiography showed severe valvular pulmonary stenosis. An ultrasound-guided fine needle aspiration of the right cervical lymph node showed papillary thyroid carcinoma. A thorough literature review using PubMed revealed no documented cases of concurrent PTC and PVS, supporting the novelty of this case. While the possibility of a coincidental coexistence cannot be excluded, this unique case raises the possibility of a shared developmental or molecular pathway-such as dysregulation of the rat sarcoma (RAS)/mitogen-activated protein kinase (MAPK) signaling cascade-linking both conditions. While a coincidental occurrence remains plausible, the overlap with pathways implicated in RASopathies warrants further investigation. Multidisciplinary evaluation was critical in planning appropriate oncologic and cardiologic management. This case underscores a rare coexistence of papillary thyroid carcinoma and severe pulmonary valve stenosis in a young adult without any identifiable congenital syndrome. Although the association may be incidental, it prompts consideration of potential shared developmental or molecular mechanisms. Importantly, the presence of severe pulmonary stenosis has significant clinical implications, contributing to symptom burden and influencing the timing and selection of therapeutic interventions, including both cardiac and oncologic management strategies.

## Introduction

Papillary thyroid carcinoma (PTC) is the most common type of thyroid malignancy, with a significant increase in the incidence in the United States from 4.6 to 17.9 per 100000 between 1975 to 2017. Despite this rising incidence, PTC typically follows an indolent course and carries a favorable prognosis [1-3]. Distant metastases occur in fewer than 10% of cases, most commonly involving the lungs and bones [4]. Cardiac metastasis, however, is exceedingly rare, reported in less than 2% of autopsy cases [5].

Pulmonary valve stenosis (PVS) is a congenital valvular abnormality characterized by obstruction of the right ventricular outflow tract. It may occur at the valvular, subvalvular, or supravalvular level, with valvular stenosis being the most frequent form. PVS accounts for approximately 7-12% of congenital heart disease and is typically diagnosed in childhood, although milder cases may remain undetected until adulthood [6,7]. While it often presents as an isolated lesion, PVS can be associated with syndromic conditions such as Noonan syndrome, congenital rubella, or tetralogy of Fallot, and in rare cases, may be acquired, for example, due to carcinoid heart disease.

To date, there are no published reports in the literature describing a co-occurrence of PTC and PVS in the same patient. This raises intriguing questions regarding the possibility of a coincidental presentation versus an under-recognized pathophysiological or genetic link. We present a rare case of a young adult male with no known congenital syndrome who was found to have both papillary thyroid carcinoma and severe pulmonary valve stenosis, highlighting the diagnostic and management challenges posed by this unusual combination.

## Case presentation

A 28-year-old male, smoker with a five-pack-year history, presented with a painless right neck swelling of 5 months duration. On physical examination, the patient appeared clinically stable. Neck examination revealed a firm, non-tender right cervical lymphadenopathy. The thyroid gland was not palpable, with no tenderness, nodularity, bruit, deviation of the trachea, or exophthalmos. Cardiovascular examination demonstrated a palpable thrill, normal first and second heart sounds, and a systolic crescendo-decrescendo murmur best heard at the left upper sternal border, which increased with inspiration. Jugular venous pressure was not elevated. There was no hepatomegaly, ascites, or peripheral edema. No syndromic features were noted. Laboratory results are shown in Table 1.

**Table 1:** Laboratory results at admission WBC: white blood cell; TSH: thyroid-stimulating hormone; PTH: parathyroid hormone; Na: sodium; K: potassium; Cl: chloride; AST: aspartate aminotransferase; ALT: alanine aminotransferase; INR: international normalized ratio; PT: prothrombin time; PTT: partial thromboplastin time.

Laboratory	Result	Reference range
WBC	9.4 × 10⁹/L	4.0 – 10.0 × 10⁹/L
Hemoglobin	16 g/dL	13.5 – 17.5 g/dL
Platelets	244 × 10⁹/L	150 – 400 × 10⁹/L
TSH	2.28 mIU/L	0.4 – 4.0 mIU/L
Free T4	0.95 ng/dL	0.8 – 1.8 ng/dL
Free T3	3.23 pg/mL	2.3 – 4.2 pg/mL
PTH	66 pg/mL	10 – 65 pg/mL
Na	136 mmol/L	135 – 145 mmol/L
K	5.1 mmol/L	3.5 – 5.2 mmol/L
Cl	102 mmol/L	98 – 107 mmol/L
Urea	4.5 mmol/L	2.5 – 7.5 mmol/L
Creatinine	81 µmol/L	62 – 106 µmol/L
AST	36 U/L	< 35 U/L
ALT	49 U/L	< 45 U/L
Total Bilirubin	18 µmol/L	5 – 21 µmol/L
Albumin	46 g/L	35 – 50 g/L
Calcium	2.41 mmol/L	2.10 – 2.60 mmol/L
INR	1.15	0.8 – 1.2
PT	15 seconds	11 – 14 seconds
PTT	22 seconds	25 – 35 seconds

**Figure 1:**
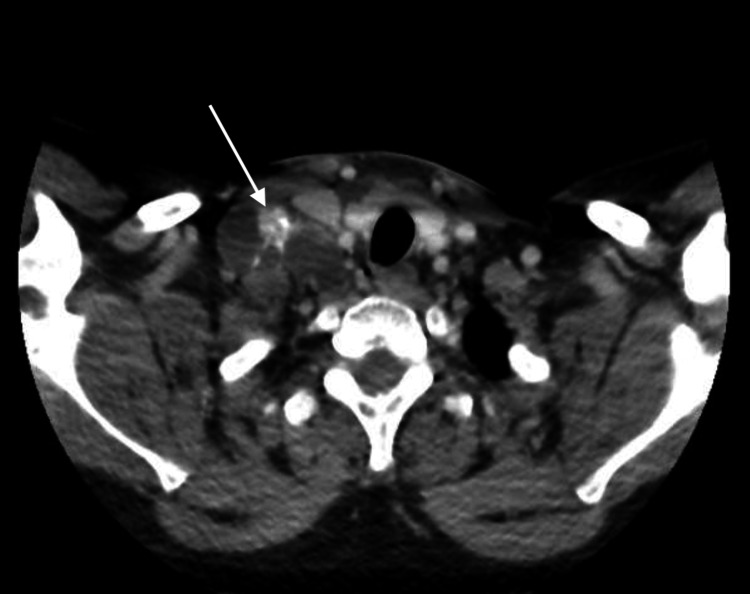
Contrast-enhanced CT neck Shows right cervical lymph node enlargement (white arrow). CT: Computed tomography.

**Figure 2:**
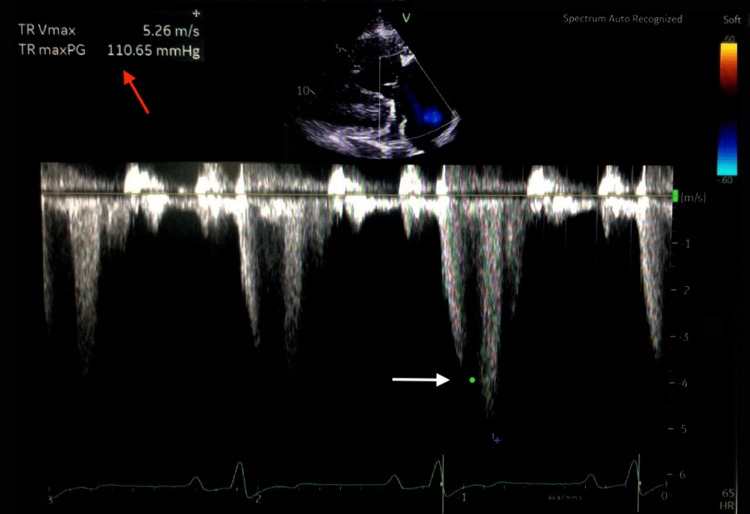
Trans-thoracic echocardiography with continuous-wave Doppler at the tricuspid valve Shows markedly elevated regurgitant jet velocity (white arrow). The calculated TRVmax was 5.26 m/s with a pressure gradient of 110 mmHg (red arrow), findings consistent with severe pulmonary hypertension. PG: pressure gradient; TR: tricuspid regurgitation; TRVmax: tricuspid regurgitation maximal velocity.

**Figure 3:**
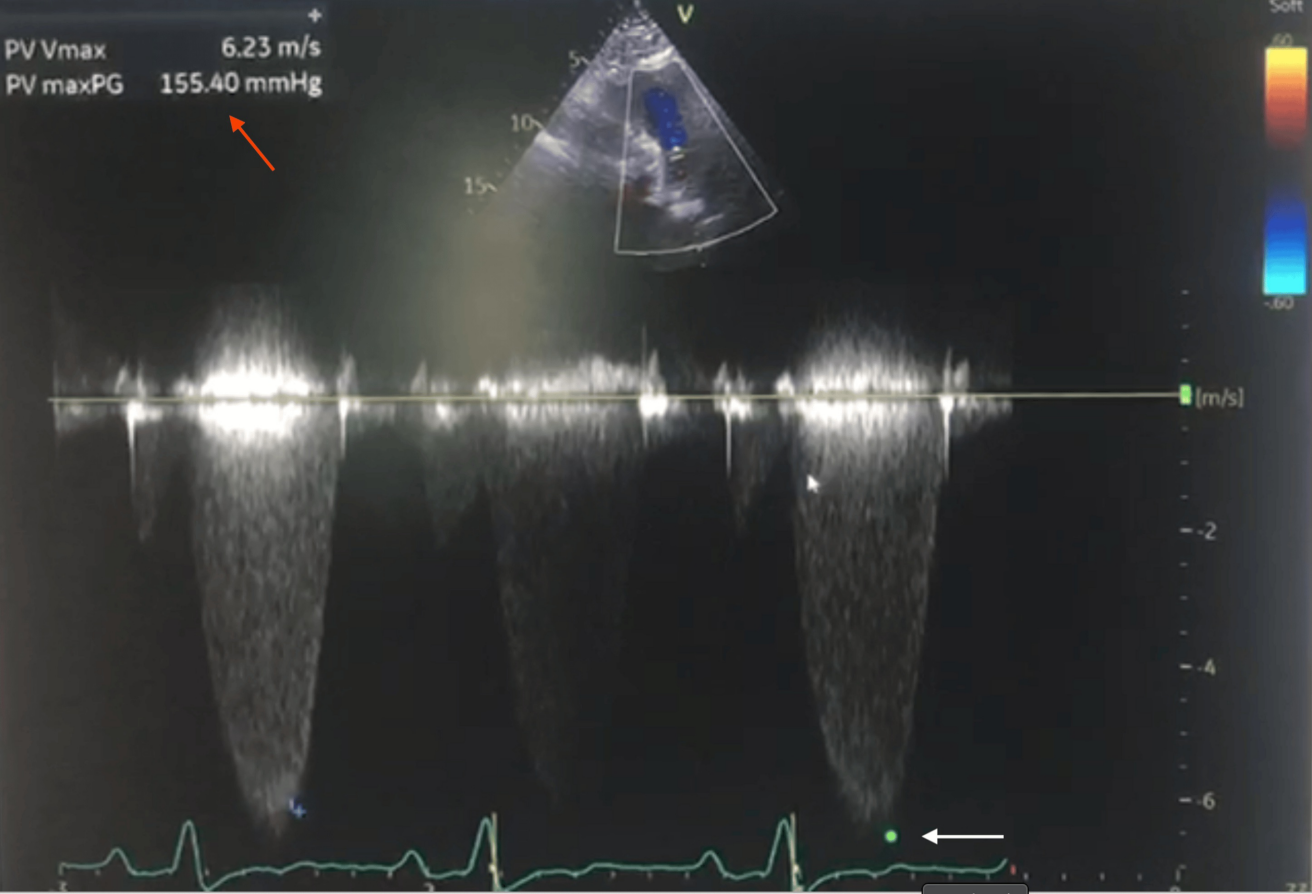
Trans-thoracic echocardiography with continuous-wave Doppler at the pulmonary valve Demonstrates markedly elevated velocity across the valve (white arrow). The calculated PV Vmax was 6.23 m/s with a peak pressure gradient of 155 mmHg (red arrow), consistent with severe valvular pulmonary stenosis. PG: pressure gradient; PV: pulmonary valve; PV Vmax: pulmonary valve maximal velocity.

Neck ultrasound showed multiple enlarged, heterogeneous, rounded right cervical lymph nodes, the largest measuring 2.9 × 2.5 cm, with internal anechoic areas and echogenic foci suggestive of necrotizing, liquefied lymph nodes with calcification. The visualized thyroid gland appeared unremarkable. CT imaging of the brain, neck, chest, abdomen, and pelvis revealed no evidence of metastases; however, cardiomegaly was noted (Figure 1). Transthoracic echocardiography demonstrated severe valvular pulmonary stenosis with a peak gradient of 155 mmHg, flattening of the interventricular septum consistent with right ventricular (RV) pressure overload, a severely dilated RV with preserved systolic function, and normal mitral and aortic valves. Left ventricular systolic function was preserved with an ejection fraction >55% (Figures 2, 3).

**Figure 4:**
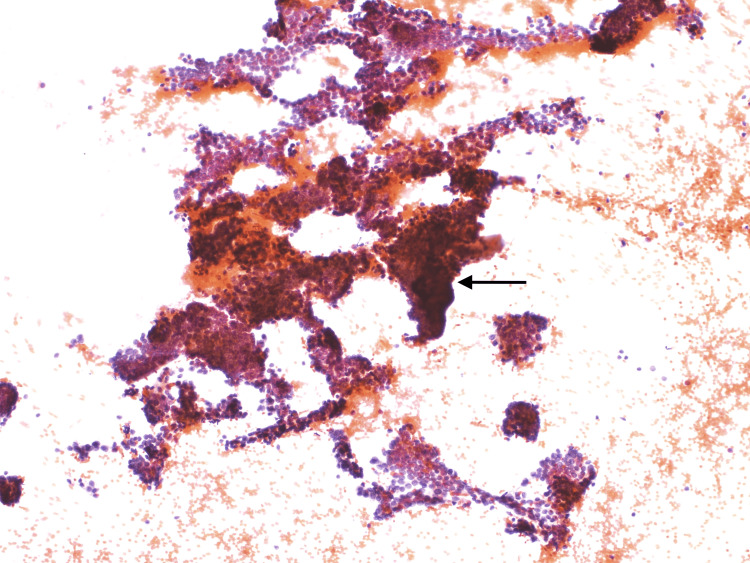
Cytology smear from a right cervical lymph node Dense cellularity with overlapping clusters of cells (black arrow)

**Figure 5:**
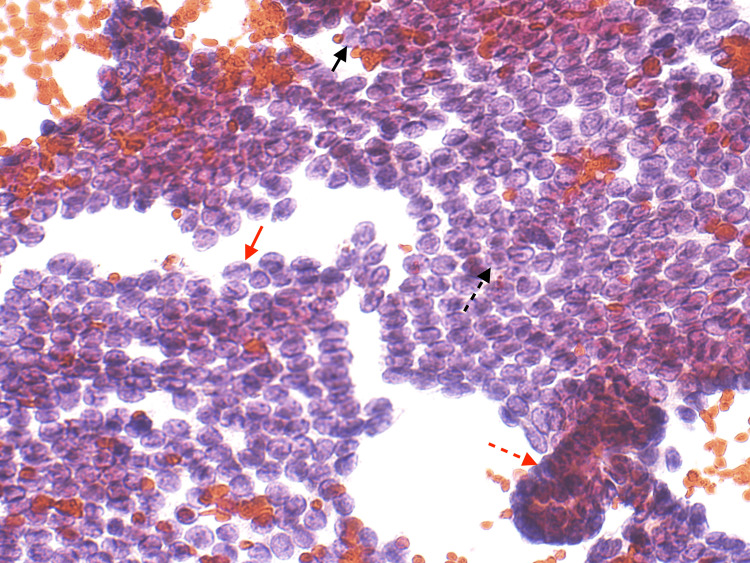
Cytology smear from the right cervical lymph node This slide shows features of papillary thyroid carcinoma, Orphan Annie eye nuclei is shown in the black arrow, nuclear grooves are shown in red arrow, intra-nuclear pseudo-inclusions are shown in dashed black arrow, and nuclear overlapping and crowding is shown dashed red arrow

Ultrasound-guided fine-needle aspiration (FNA) of the right cervical lymph node was performed, and cytology was diagnostic of papillary thyroid carcinoma (Figures 4, 5).

Multidisciplinary consultations were obtained with otolaryngology, endocrinology, and cardiology. The patient was subsequently planned for total thyroidectomy.

## Discussion

Papillary thyroid carcinoma (PTC) is generally associated with a favorable prognosis and slow progression. While regional lymph node metastasis is common, distant metastasis occurs in fewer than 10% of cases, with the lungs and bones being the most frequent sites [4]. Cardiac involvement from thyroid malignancies is exceedingly rare, with an estimated incidence of 0-2% in autopsy series [5]. In our patient, although no cardiac metastasis was detected on imaging, the incidental finding of cardiomegaly prompted further evaluation, leading to the diagnosis of severe pulmonary valve stenosis (PVS).

PVS is a congenital cardiac lesion that typically manifests during infancy or early childhood. Adult presentations, although uncommon, have been reported, particularly in patients with mild or compensated forms of the disease [6]. Remarkably, our patient was asymptomatic despite a severe pressure gradient (110 mmHg), likely reflecting chronic right ventricular adaptation. Nevertheless, the echocardiographic findings of a markedly dilated right ventricle and interventricular septal flattening confirm significant hemodynamic compromise.

Interestingly, the thyroid gland appeared sonographically normal, and fine-needle aspiration confirmed PTC from the cervical lymph node. This raises two possible explanations: (1) the malignant lesion could have arisen from ectopic thyroid tissue in the cervical region, or (2) the primary thyroid tumor may have been a small nodule undetectable by conventional ultrasound.

A comprehensive literature review using PubMed revealed no documented cases of concurrent PTC and PVS, supporting the novelty of this case. While the possibility of a coincidental coexistence cannot be excluded. The coexistence of papillary thyroid carcinoma (PTC) and severe pulmonary valve stenosis (PVS) in a young adult without syndromic features is particularly intriguing. Noonan syndrome has been reported to link cardiac malformations, including PVS, with endocrine dysfunction or thyroid dysgenesis [8]. In Noonan syndrome, PVS, which is often linked to dysplastic valves, occurs in up to 50-60% of affected individuals, making it the most common cardiac manifestation of the syndrome [8]. In pediatric populations with isolated PVS, approximately 9% are eventually diagnosed with a Noonan spectrum disorder [9]. At the molecular level, pathogenic variants in rat sarcoma (RAS)- mitogen-activated protein kinase (MAPK) pathway genes, such as protein tyrosine phosphatase non-receptor type 11 (PTPN11), son of sevenless homolog 1 (SOS1), rapidly accelerated fibrosarcoma 1 (RAF1), and v-raf murine sarcoma viral oncogene homolog B1 (BRAF), have been implicated, linking dysregulated signaling to concurrent cardiac and developmental anomalies [10]. While some of these variants (e.g., PTPN11) also confer increased risk of malignancy, including leukemia, there are no documented cases where PTC co-occurs with PVS, whether syndromic or sporadic [8-10]. This suggests a potentially novel or coincidental presentation. In this context, genetic testing-even in the absence of overt clinical features-could be invaluable for ruling out subtle RASopathy and clarifying a possible shared etiology.

From a clinical perspective, the presence of significant cardiac disease has important implications for surgical and anesthetic planning in patients undergoing thyroidectomy. Preoperative cardiology assessment and potential correction or stabilization of the valvular lesion may be warranted to minimize perioperative risks. In our case, multidisciplinary collaboration between endocrinology, otolaryngology, and cardiology teams was essential to ensure optimal care.

## Conclusions

This case underscores a rare coexistence of papillary thyroid carcinoma and severe pulmonary valve stenosis in a young adult without any identifiable congenital syndrome. Although the association may be incidental, it prompts consideration of potential shared developmental or molecular mechanisms, which highlights the value of genetic testing in this case. Importantly, the presence of severe pulmonary stenosis has significant clinical implications, contributing to symptom burden and influencing the timing and selection of therapeutic interventions, including both cardiac and oncologic management strategies.

## References

[1] Haugen BR, Alexander EK, Bible KC (2016). 2015 American Thyroid Association management guidelines for adult patients with thyroid nodules and differentiated thyroid cancer: The American Thyroid Association Guidelines Task Force on thyroid nodules and differentiated thyroid cancer. Thyroid.

[2] Lim H, Devesa SS, Sosa JA, Check D, Kitahara CM (2017). Trends in thyroid cancer incidence and mortality in the United States, 1974-2013. JAMA.

[3] LeClair K, Bell KJ, Furuya-Kanamori L, Doi SA, Francis DO, Davies L (2021). Evaluation of gender inequity in thyroid cancer diagnosis: differences by sex in US thyroid cancer incidence compared with a meta-analysis of subclinical thyroid cancer rates at autopsy. JAMA Intern Med.

[4] Nixon IJ, Whitcher MM, Palmer FL (2012). The impact of distant metastases at presentation on prognosis in patients with differentiated carcinoma of the thyroid gland. Thyroid.

[5] Giuffrida D, Gharib H (2001). Cardiac metastasis from primary anaplastic thyroid carcinoma: report of three cases and a review of the literature. Endocr Relat Cancer.

[6] Pandya B, Cullen S, Walker F (2016). Congenital heart disease in adults. BMJ.

[7] Samánek M, Slavík Z, Zborilová B, Hrobonová V, Vorísková M, Skovránek J (1989). Prevalence, treatment, and outcome of heart disease in live-born children: a prospective analysis of 91,823 live-born children. Pediatr Cardiol.

[8] Roberts AE, Allanson JE, Tartaglia M, Gelb BD (2013). Noonan syndrome. Lancet.

[9] Bell JM, Considine EM, McCallen LM, Chatfield KC (2021). The prevalence of Noonan spectrum disorders in pediatric patients with pulmonary valve stenosis. J Pediatr.

[10] Sun L, Xie YM, Wang SS, Zhang ZW (2022). Cardiovascular abnormalities and gene mutations in children with noonan syndrome. Front Genet.

